# α- and β-Genotyping of Thalassemia Patients Based on a Multimodal Liver MRI Radiomics Model: A Preliminary Study in Two Centers

**DOI:** 10.3390/diagnostics13050958

**Published:** 2023-03-03

**Authors:** Fengming Xu, Qing Feng, Jixing Yi, Cheng Tang, Huashan Lin, Bumin Liang, Chaotian Luo, Kaiming Guan, Tao Li, Peng Peng

**Affiliations:** 1 Department of Radiology, The First Affiliated Hospital of Guangxi Medical University, Nanning 530021, China; 2 NHC Key Laboratory of Thalassemia Medicine, Guangxi Medical University, Nanning 530021, China; 3 Department of Radiology, Fourth Affiliated Hospital of Guangxi Medical University, Liuzhou Worker’s Hospital, Liuzhou 545005, China; 4 Department of Pharmaceutical Diagnosis, GE Healthcare, Changsha 410005, China; 5 School of International Education, Guangxi Medical University, Nanning 530021, China

**Keywords:** radiomics, thalassemia, genotype, magnetic resonance imaging, model

## Abstract

Background: So far, there is no non-invasive method that can popularize the genetic testing of thalassemia (TM) patients on a large scale. The purpose of the study was to investigate the value of predicting the α- and β- genotypes of TM patients based on a liver MRI radiomics model. Methods: Radiomics features of liver MRI image data and clinical data of 175 TM patients were extracted using Analysis Kinetics (AK) software. The radiomics model with optimal predictive performance was combined with the clinical model to construct a joint model. The predictive performance of the model was evaluated in terms of AUC, accuracy, sensitivity, and specificity. Results: The T2 model showed the best predictive performance: the AUC, accuracy, sensitivity, and specificity of the validation group were 0.88, 0.865, 0.875, and 0.833, respectively. The joint model constructed from T2 image features and clinical features showed higher predictive performance: the AUC, accuracy, sensitivity, and specificity of the validation group were 0.91, 0.846, 0.9, and 0.667, respectively. Conclusion: The liver MRI radiomics model is feasible and reliable for predicting α- and β-genotypes in TM patients.

## 1. Introduction

Thalassemia (TM) is a common autosomal recessive genetic disease caused by defective globin chain production, which mainly occurs in Mediterranean populations, Africa, the Middle East, Central Asia, India, Southern China, and the Far East [1]. Due to population migration, nowadays, TM is also common in many immigration countries around the world [1,2,3]. The disease types are classified as α-, β-, δβ-, and δβγ-TM based on the specific globin chains with defective synthesis. In terms of epidemiology, α-and β-TM are the most common important genotypes [4]. Excessive generation of α-or β-globin chains may lead to ineffective erythropoiesis, premature erythroid destruction, and anemia. TM is generally asymptomatic in trait and carrier states [3]. There are significant differences in clinical signs, medical care, and quality of life among patients with different TM genotypes. Severe α-thalassemia causes fetal oedema and is usually fatal at birth. Severe β-thalassemia requires lifelong blood transfusion from early childhood (usually before two years of age). The manifestations of intermedia α- and β-thalassemia are different due to gene mutations or deletions, but the severe form causes symptomatic anemia and requires blood transfusion [2,3,4]. Of particular note is that Hb Bart’s hydrops fetalis is the most severe type of α-TM and usually results in death. The baby usually dies during pregnancy (23–38 weeks) or shortly after delivery (unless treated with intrauterine blood transfusions). β-TM and related diseases caused by hemoglobin variants, such as hemoglobin S and hemoglobin E, are more prevalent due to their severe consequences. β-TM is clinically more important than α-TM and requires ongoing medical care [5,6]. Complications of thalassemia are mainly caused by bone marrow expansion, extramedullary hematopoiesis, and iron deposition in peripheral tissues. The effects of these complications include diseases of the skeletal system, endocrine organs, heart, and liver [7]. In the past 50 years, with the popularity of transfusion and iron chelation therapy and the improvement of iron-overload monitoring [3], genetic counseling and screening for high-risk groups have helped to reduce the prevalence of TM [1,3].The traditional genetic diagnosis method for TM gene carriers or patients is to determine the TM-related phenotypic characteristics of the group through hematological and biochemical tests and subsequent molecular genetic tests [8]. Standard detection methods require peripheral blood sampling, amniocentesis, and conventional chorionic villus sampling (CVS) [9]. Peripheral blood sampling is invasive, while CVS and amniocentesis are not only invasive but are also associated with abortion risks as high as 1/200–400 and 1/100–200, respectively [10]. To the best of our knowledge, there are no widely available assays that can be performed non-invasively for genotyping TM patients.

Magnetic resonance imaging (MRI) technology has become an important non-invasive examination tool for the diagnosis and evaluation of many diseases. MRI has been widely accepted as the primary method for non-invasive determination of liver iron concentration for the assessment of related complications in TM patients, especially for regular monitoring of organ iron load, quantification of the degree of iron load in different organs, and assessment of iron-removal effect [11,12]. With the continuous development of medical imaging technology and radiomics, macroscopic image data can be deeply studied to reflect many advanced data that cannot be recognized by the naked eye. Many studies have shown that radiomics technology can be applied in the differential diagnosis of many diseases, the prediction of tumor metastasis, the prediction of the existence of specific gene mutations, etc., and it has shown good predictive performance [13,14,15,16].

This study aimed to explore the predictive value of an MRI radiomics model, a non-invasive method for predicting genotypes, for the genotyping of TM patients by constructing a prediction model for α- and β-genotypes in TM patients. Since different organs in TM patients have different rates of complications in the whole course of the disease and since the liver is the primary organ involved in related complications (such as iron overload) [17,18], this study took the liver as the target organ for preliminary exploration. 

## 2. Materials and Methods

### 2.1. Clinical Data

**Figure 1:**
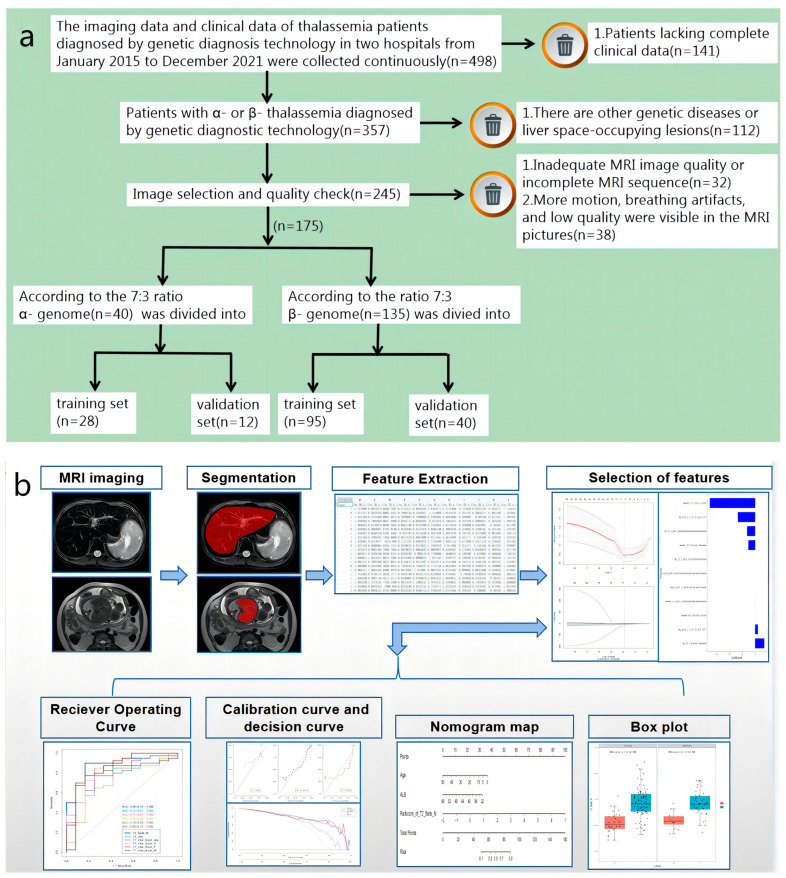
(**a**) A simplified flow chart of the patient selection process. (**b**) A simplified flow chart for constructing the MRI image feature model to predict α-and β-genotypes in TM patients.

This study was approved by the ethics committees of two hospitals: no. 2022-ky-e-191 and no. LW2022057. Due to the retrospective nature of the study, informed consent was waived. The imaging data and clinical data of 498 TM patients diagnosed by genetic diagnosis technology in the two hospitals from January 2015 to December 2021 were continuously collected. Inclusion criteria: (1) thalassemia patients with a single genotype (α or β) diagnosed by genetic diagnosis technology; (2) complete liver MRI plain scan image sequence, including T2 (T2 fblade fs/T2 ssfse tra bh), T2* (multi-echo GRE T2*), and T1 opp/in/F/W (T1 vibe dixon opp/in/F/W); (3) complete clinical data are recorded, including age, gender, liver T2* value, serum ferritin, blood routine, and liver and kidney function indexes of the patient, within one week after MRI examination (liver T2* value, serum ferritin, and blood routine test results were only used as baseline data); (4) regular or irregular blood transfusion treatment; (5) regular or irregular iron-removal treatment. Exclusion criteria: (1) there are macroscopic liver space-occupying lesions; (2) MRI sequence is incomplete, or image quality does not meet the diagnostic requirements. Figure 1a shows the simplified processes of the inclusion and exclusion approaches in this study. A total of 175 TM patients were finally included in this study (including 5 fetuses), including 123 (70.29%) in the training set and 52 (29.71%) in the validation set, including 101 males (57.71%) and 74 females (42.29%), aged 4–50 years. (Median age was 10 years old, and the gestational ages of the 5 fetuses were not included. The gestational ages of the five fetuses were 234 days, 218 days, 242 days, 248 days, and 228 days, respectively.) As confirmed by genetic diagnosis, there were 40 cases in the α-genome group (28 cases in the training set and 12 cases in the test set) and 135 cases in the β-genome group (95 cases in the training set and 40 cases in the test set). 

### 2.2. MRI Scanning Method

All MRI studies were performed using a 3.0 T scanner (Verio, Siemens Healthcare, Erlangen, Germany) with 18-channel abdominal phased-array surface coils and 32-channel integrated spinal matrix coils. The examiner’s position was head advanced and supine. GRE sequences were acquired at the end of a single breath-hold, and a single-slice slice was obtained from the largest cross-sectional area of the liver. The remaining sequences scanned the entire liver. GRE sequence: TR = 200 ms, TE = 0.97 ms, 2.38 ms, 3.79 ms, 5.20 ms, 6.61 ms, 8.02 ms, 9.43 ms, 10.84 ms, 12.25 ms, 13.66 ms, 15.07 ms, 16.48 ms; turn angle = 20°, matrix = 64 × 128, FOV = 200 mm × 400 mm, layer thickness = 10 mm. T2 sequence: TR = 3110 ms, TE = 87 ms; turn angle = 104°, matrix = 384 × 384, FOV = 380 mm × 380 mm, layer thickness = 5 mm. T1 vibe dixon (opp/in/F/W): TR = 6.3 ms, TE = 2.39 ms, 4.77 ms; turn angle = 9°, matrix = 208 × 256, FOV = 308.8 mm × 380 mm, layer thickness = 4.5 mm.

### 2.3. Screening of Clinical Features

In the training group, univariate logistic regression analysis was used to retain the clinical features that were statistically significant for the identification of α- and β-genotypes in thalassemia patients (*p* < 0.05). For the clinical features with statistical significance, multivariate logistic regression analysis was used; the clinical features that were still statistically significant (*p* < 0.05) were retained, and a clinical training set model was constructed.

### 2.4. Radiomics Analysis

**Figure A1:**
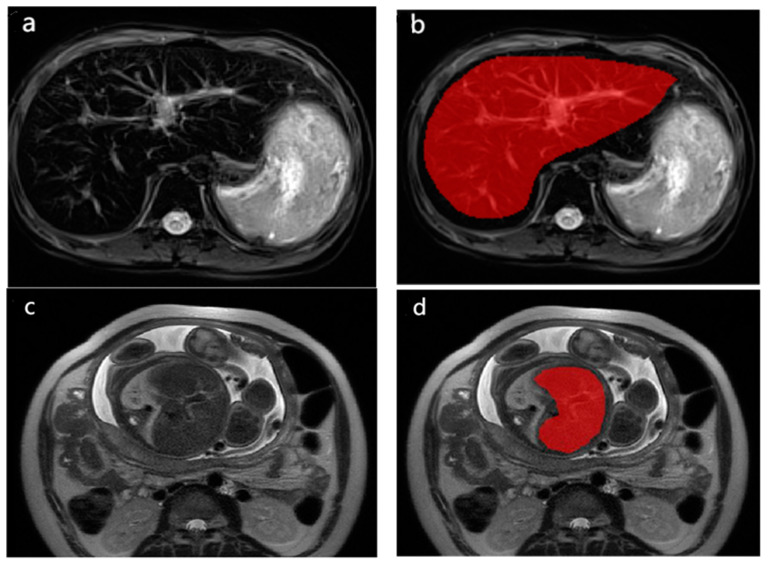
(**a**) (Adult) is the original MRI image of the largest cross section of the liver T2 sequence. (**b**) (Adult) is a sequence image of liver T2 manually segmented by ITK-SNAP. (**c**) (Fetus) is the original MRI image of the largest cross section of the liver T2 sequence. (**d**) (Fetus) is a sequence image of liver T2 manually segmented by ITK-SNAP.

MRI scan images of all patients were exported from the PACS system workstation in DICOM format. The radiomics analysis included the following steps: (1) region of interest (ROI) two-dimensional segmentation: ROI segmentation was performed using ITK-SNAP software (version 3.6.0, Philadelphia, PA, USA). Before performing ROI segmentation, the “Auto” function in ITK-SNAP was used to adjust the image window width and window level. The first echo time was selected for the T2* image, and the entire liver cross section was manually segmented. The largest liver cross section was selected for manual segmentation for all remaining sequence images (Figure A1, Appendix A). The segmented images were called “NIfTI-Format”. (2) Feature extraction: data were imported into AK (V3.2.0, Workbench2014, GE Healthcare) analysis software to extract image radiomics features, including first-order features, shape (Shape), grayscale co-occurrence matrix (GLCM), grayscale run-length matrix (GLRLM), grayscale size zone matrix (GLSZM), grayscale dependency matrix (GLDM), and neighborhood grayscale features of difference matrix (NGTDM). The selected image transforms were: logarithmic transform (LoG), parameter Sigma select 2.0, 3.0; wavelet transform (Wavelet), Level 1; local binary mode (LBP), Level 2, Radius 1.0, Subdivision select 1. A total of 1316 features were extracted for each sequence model. (3) Radiomics feature selection and classifier construction: the 40 α-gene patients and 135 β-gene patients were divided into a training group (28 α-genes, 95 β-genes) and a validation group (12 α-genes, 40 β-genes) at a ratio of 7:3 [19]. First, in the training group, Max-Relevance and Min-Redundancy (mRMR) were used for the first feature dimensionality reduction, removing features with *p* > 0.05, and retaining the best 20 features. Then, the Least Absolute Shrinkage and Selection Operator (LASSO) model was used to further screen out the optimal features and establish a final logistic regression classifier for the training datasets for radiometric feature selection. (This method is a kind of compression estimation. It obtains a more refined model by constructing a penalty function, making it compress some regression coefficients, that is, the sum of the absolute values of the force coefficients is less than a certain fixed value. Some regression coefficients were also set to zero. Therefore, it retains the advantage of subset shrinkage and is a biased estimator dealing with data with complex collinearity [19,20].) Ten-fold cross validation was used to control the stringency of the constraint on the sum of the absolute values of the regression coefficients. In our codes, the LASSO algorithm contains a 10-fold cross validation, which was used for proving the reliability of the radiomics training model. Finally, a logistic regression model was used to establish radiomics scores (Rad scores), and the training and validation datasets were calculated. Figure 1b shows a simplified flow chart of the radiomics model.

### 2.5. Statistical Analysis

Statistical analysis of clinical data was performed using SPSS 26.0 software. The Kolmogorov–Smirnov test was used to test whether the measurement data conformed to a normal distribution, and *p* > 0.05 indicated that the data conformed to a normal distribution. Measurement data with a normal distribution were expressed as means ± standard deviations ($\bar{x}\pm s$); measurement data that did not meet the normal distribution were expressed as medians (M) and interquartile ranges (P_25%_–P_75%_).

### 2.6. Performance of Radiomics Prediction Models

The Hosmer–Lemeshow test was used to test the model fit. When *p* > 0.05, there was no statistically significant difference, indicating a good degree of fit. Calibration curves were used to assess whether the model-predicted probability was close to the true probability. A decision curve was used to evaluate the profitability of the model. Box plots were used to analyze the distribution differences of Rad scores in different models. The receiver operating characteristic curve (ROC), area under the curve (AUC), accuracy, sensitivity, specificity, and so on, were used to comprehensively evaluate the predictive efficiency of the model. *p* < 0.05 was considered statistically significant.

### 2.7. Intra-Observer and Inter-Observer Consistency

To detect whether there was an effect of the manual measurements on the experimental results, the intraclass correlation coefficient (ICC) was used to evaluate intra-observer and inter-observer consistency in feature extraction. At a ratio of 7:3, a total of 30 T2 images were randomly selected from the training set (*n* = 21) and the validation set (*n* = 9) for ROI segmentation and feature extraction. ROI segmentation was performed independently by two experienced radiologists. Intra-observer ICC was calculated by comparing the extracted features of Observer A (with 5 years of experience in abdominal MRI diagnosis) twice. Inter-observer ICC was calculated by comparing features extracted by Observer B (5 years of experience in abdominal MRI diagnosis) with those extracted by Observer A. When ICC > 0.75 and *p* < 0.05, consistency was considered good. All image segmentation tasks were averaged and randomly assigned to observers A and B. 

## 3. Results

### 3.1. Clinical Features

**Figure 2:**
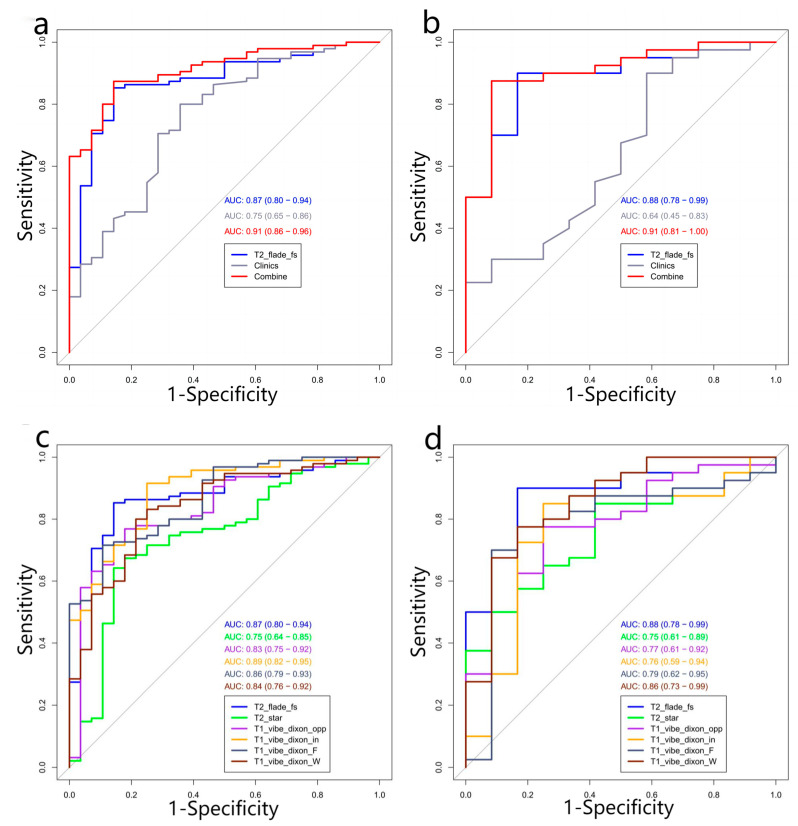
(**a**,**b**) ROC curves of α- and β-genotyping of thalassemia patients in the T2 model, clinical model, and joint model. (**c**,**d**) ROC curves of each radiomics model. Note—T2 flade fs = T2 fblade fs/T2 ssfse tra bh.

A total of 175 patients with TM (median age, 10 years; interquartile range [P_25%_–P_75%_], 7–13 years; 74 females, 101 males) were included in this study. The measurement data did not follow a normal distribution (*p* < 0.05). The liver T2* value, serum ferritin, and baseline blood routine data are shown in Table 1. The results of univariate logistic regression analysis of other clinical data are shown in Table 2. After re-screening by multivariate logistic regression, two optimal clinical features were finally included in the clinical model (Table 3). The ROC curves of the clinical model are shown in Figure 2a,b, “Clinic” curves. The AUC, accuracy, sensitivity, and specificity of the validation group were 0.64, 0.712, 0.8, and 0.417, respectively (Table 4). 

### 3.2. Radiomics Features

**Figure 3:**
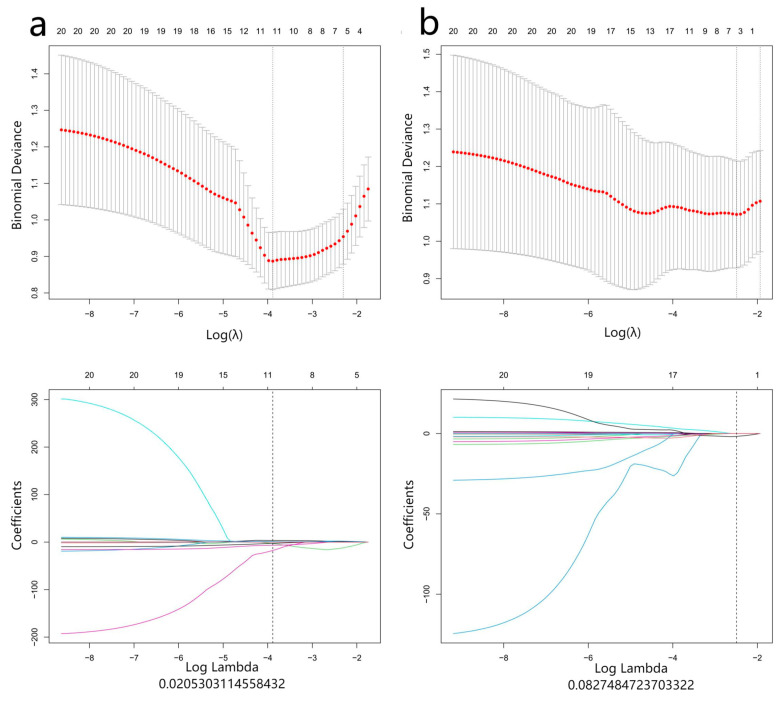

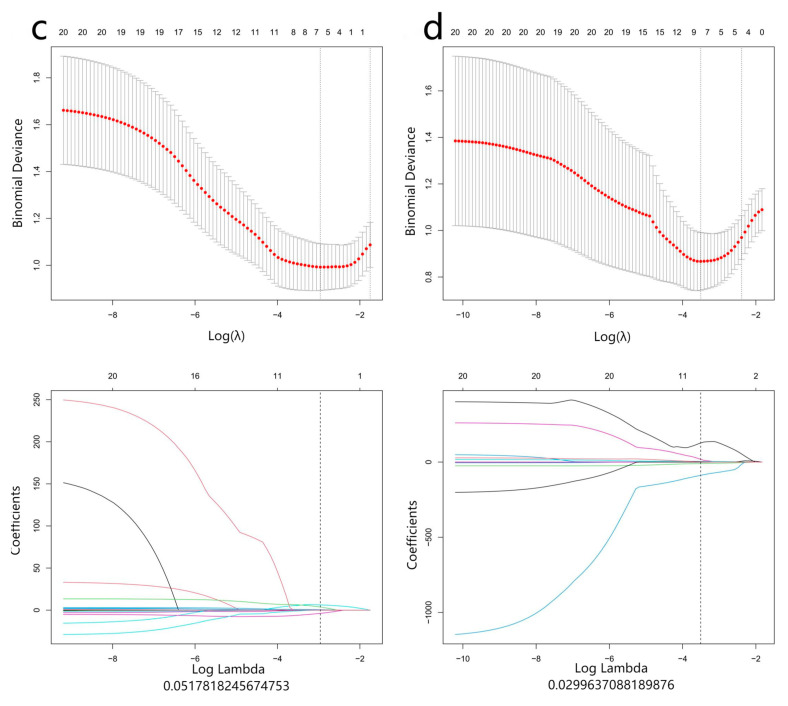

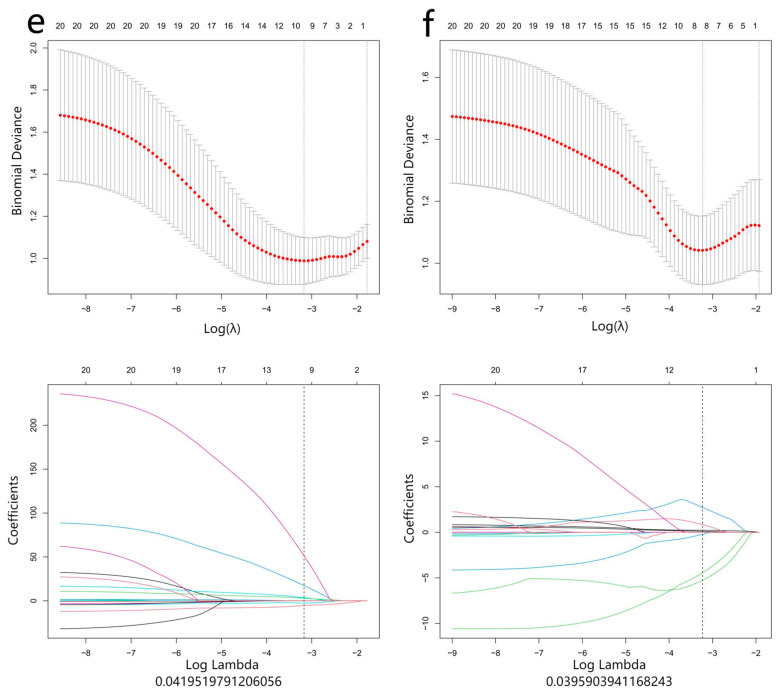
Radiomics feature dimensionality reduction LASSO coefficient distribution map ((**a**–**f**) correspond to the T2, T2*, T1 vibe dixon opp, T1 vibe dixon in, T1 vibe dixon F, and T1 vibe dixon W models, respectively). Each curve represents the change trajectory of the independent variable coefficient corresponding to different penalty coefficients, and the dotted lines are the minimum penalty coefficients.

**Figure 4:**
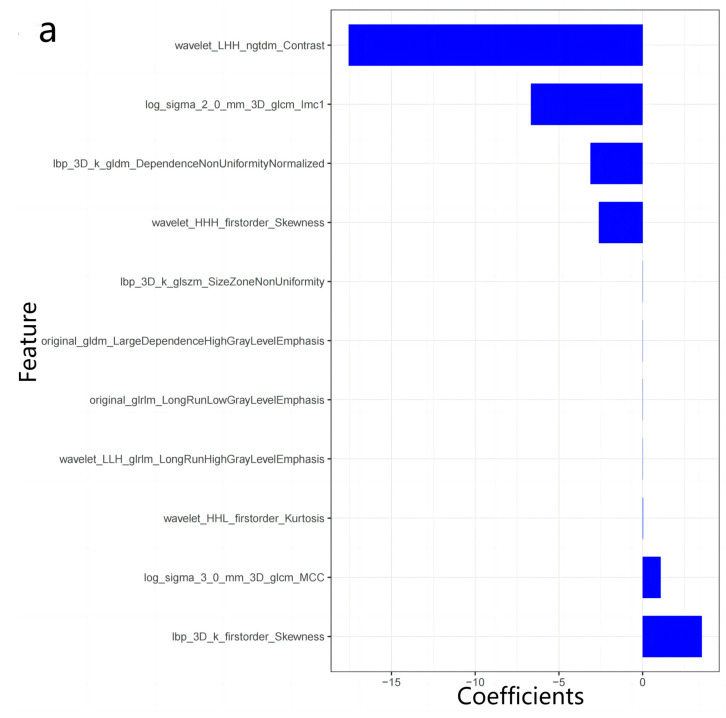

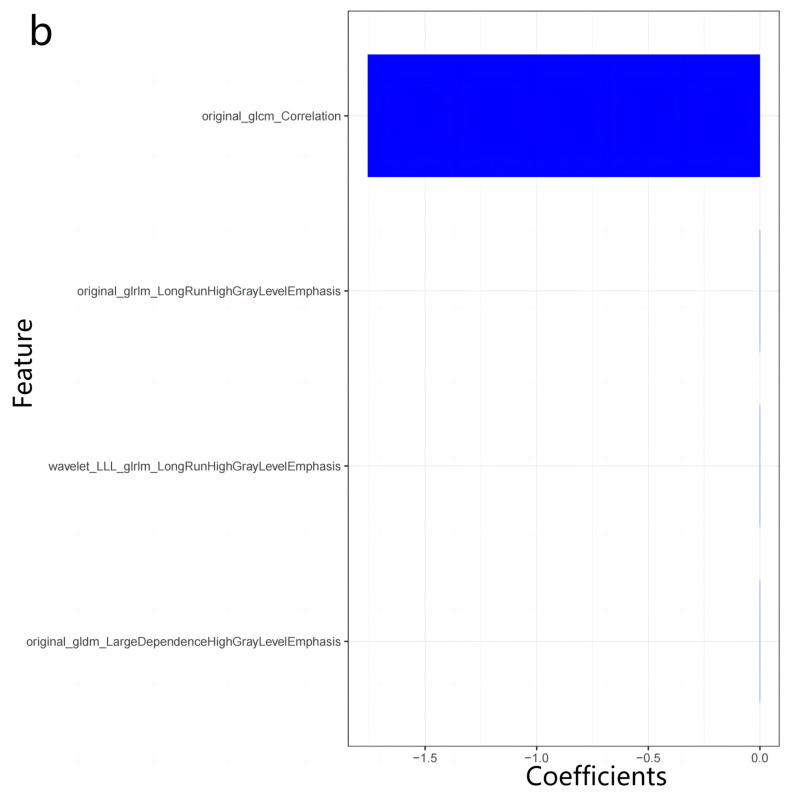

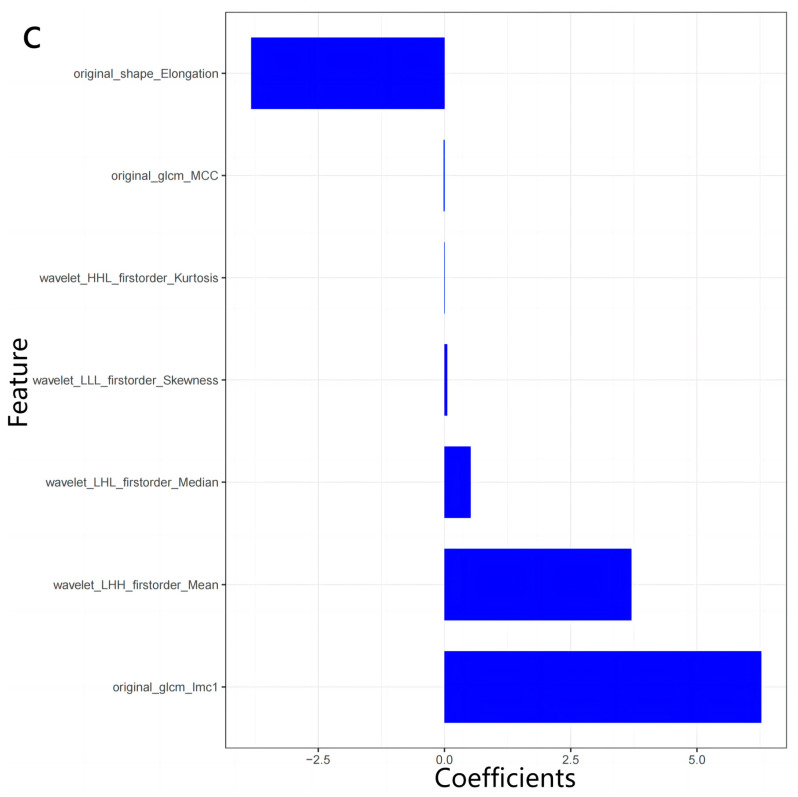

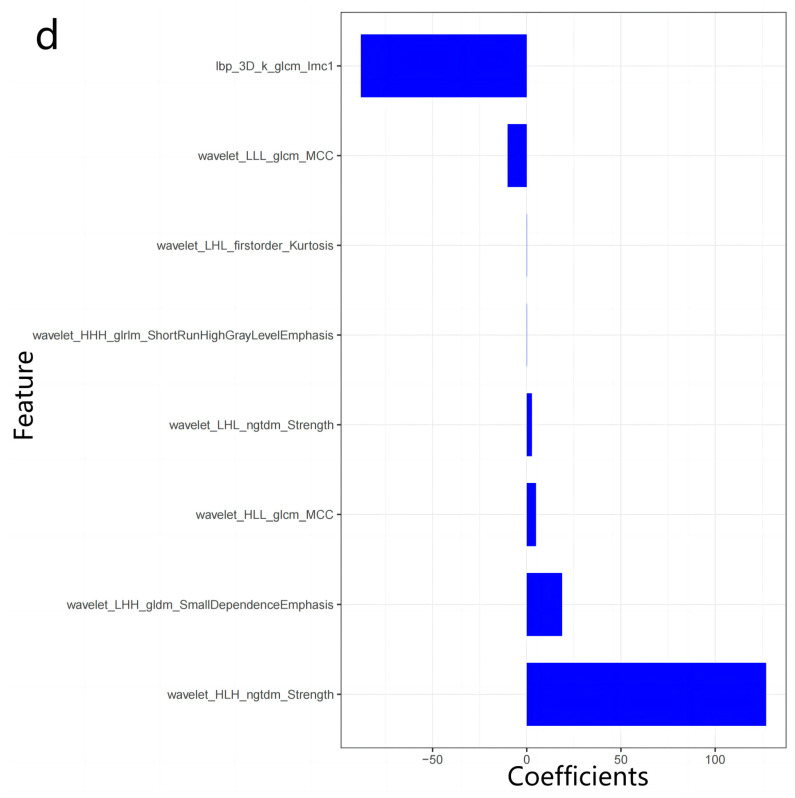

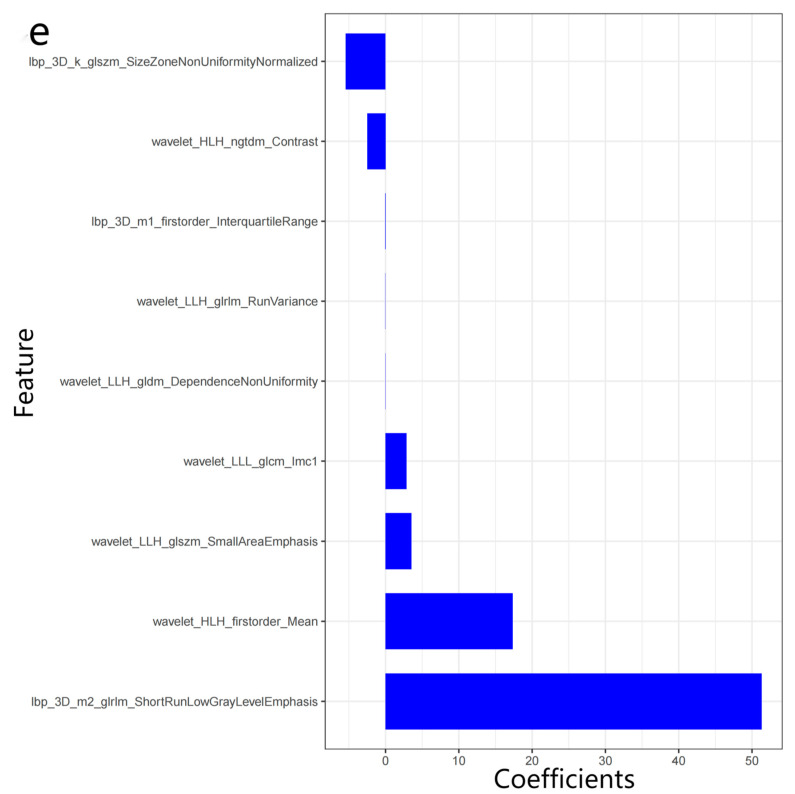

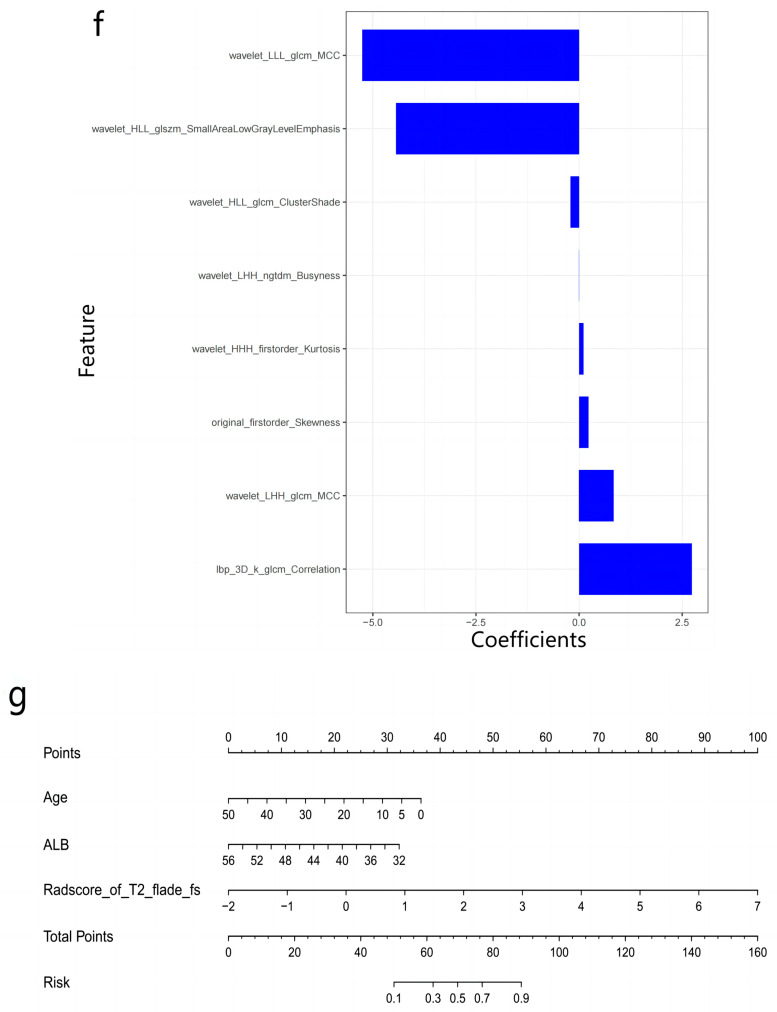
The best omics features of different image omics models are as follows: T2 model Rad score (**a**) = 3.53346224020733 × lbp_3D_k_firstorder_Skewness + 1.07465494102622 × log_sigma_3_0_mm_3D_glcm_MCC + 1.78127454519169 × 10^−2^ × wavelet_HHL_firstorder_Kurtosis + 8.36953869311654 × 10^−4^ × wavelet_LLH_glrlm_LongRunHighGrayLevelEmphasis + 7.22569069179091 × 10^−4^ × original_glrlm_LongRunLowGrayLevelEmphasis − 1.77115252453958 × 10^−3^ × original_gldm_LargeDependenceHighGrayLevelEmphasis − 7.0101953054909 × 10^−3^ × lbp_3D_k_glszm_SizeZoneNonUniformity − 2.62091113931379 × wavelet_HHH_firstorder_Skewness − 3.11332596350631 × lbp_3D_k_gldm_DependenceNonUniformityNormalized − 6.20776724661879 × (Intercept) − 6.66761355299743 × log_sigma_2_0_mm_3D_glcm_Imc1 − 17.566640428635 × wavelet_LHH_ngtdm_Contrast. T2* model Rad-score; (**b**) = 2.50144266068886 × (Intercept) − 1.24913107460922 × 10^−5^ × original_gldm_LargeDependenceHighGrayLevelEmphasis − 6.24916156246217 × 10^−5^ × wavelet_LLL_glrlm_LongRunHighGrayLevelEmphasis − 4.08064312178882 × 10^−4^ × original_glrlm_LongRunHighGrayLevelEmphasis − 1.75768099910874 × original_glcm_Correlation. T1 vibe dixon opp model Rad-score; (**c**) = 6.27425742182574 × original_glcm_Imc1 + 5.37994776377167 × (Intercept) + 3.70616662130978 × wavelet_LHH_firstorder_Mean + 0.516626652535698 × wavelet_LHL_firstorder_Median + 0.0565285647399182 × wavelet_LLL_firstorder_Skewness − 0.00597233533241726 × wavelet_HHL_firstorder_Kurtosis − 0.0176135434925847 × original_glcm_MCC-3.83246459948815 × original_shape_Elongation. T1 vibe dixon in model Rad-score; (**d**) = 127.086320720469 × wavelet_HLH_ngtdm_Strength + 18.667830611436 × wavelet_LHH_gldm_SmallDependenceEmphasis + 5.72164956963507 × (Intercept) + 4.87874941478956 × wavelet_HLL_glcm_MCC + 2.65747466451841 × wavelet_LHL_ngtdm_Strength + 0.087457883090257 × wavelet_HHH_glrlm_ShortRunHighGrayLevelEmphasis + 0.0356125450830294 × wavelet_LHL_firstorder_Kurtosis − 10.1682627437812 × wavelet_LLL_glcm_MCC-88.0170431265614 × lbp_3D_k_glcm_Imc1. T1 vibe dixon F model Rad-score; (**e**) = 51.3219769208571 × lbp_3D_m2_glrlm_ShortRunLowGrayLevelEmphasis + 17.3682101660547 × wavelet_HLH_firstorder_Mean + 3.95718718046264 × (Intercept) + 3.52431026532541 × wavelet_LLH_glszm_SmallAreaEmphasis + 2.87681895035553 × wavelet_LLL_glcm_Imc1 − 9.7943125275996×10^−5^ × wavelet_LLH_gldm_DependenceNonUniformity − 0.0338412742628526 × wavelet_LLH_glrlm_RunVariance − 5.8097236730315 × 10^−2^ × lbp_3D_m1_firstorder_InterquartileRange − 2.49038445966763 × wavelet_HLH_ngtdm_Contrast − 5.42813421361219 × lbp_3D_k_glszm_SizeZoneNonUniformityNormalized. T1 vibe dixon W model Rad-score; (**f**) = 5.26762029862077 × (Intercept) + 2.73411114054784 × lbp_3D_k_glcm_Correlation + 0.839563442800306 × wavelet_LHH_glcm_MCC + 0.227753966581487 × original_firstorder_Skewness + 0.106672491336863 × wavelet_HHH_firstorder_Kurtosis − 2.23550830619043×10^−4^ × wavelet_LHH_ngtdm_Busyness − 0.205280111379976 × wavelet_HLL_glcm_ClusterShade − 4.44029239333195 × wavelet_HLL_glszm_SmallAreaLowGrayLevelEmphasis − 5.25648829872901 × wavelet_LLL_glcm_MCC. Nomogram map of the joint model constructed from T2 image features and clinical features is as Figure 4g.

**Figure 5:**
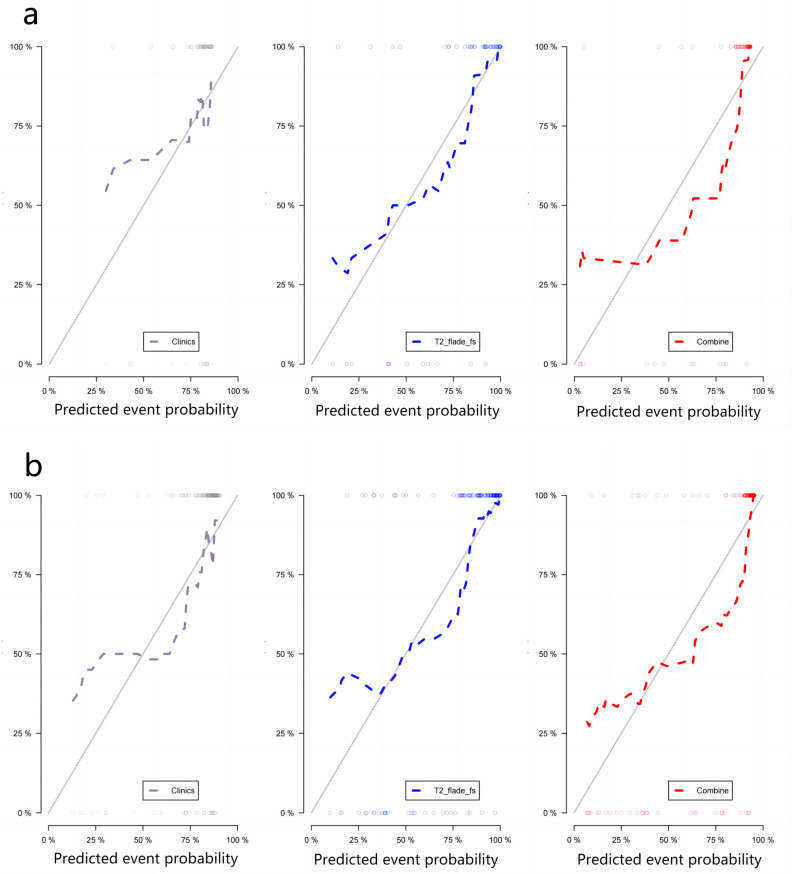

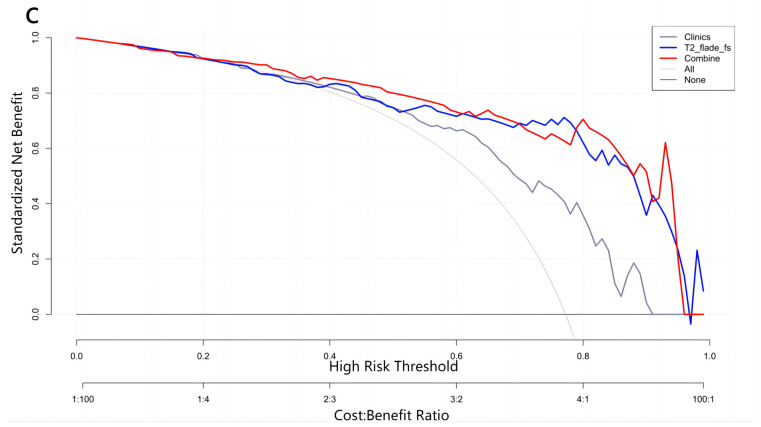
Calibration curve and decision curve analysis. The calibration curves of the training group (**a**) and the validation group (**b**) prove that the Rad scores of the T2 model, the clinical model, and the joint model have good fitness. (**c**) Decision curve analysis of the T2 model, the clinical model, and the joint model. The Y-axis represents the net benefit, which is calculated by adding up the benefits (gaining true positives) and subtracting the weighted harms (deleting false positives). A model is considered the best method for feature selection if it has the highest net benefit. Note—T2 flade fs = T2 fblade fs/T2 ssfse tra bh.

**Figure 6:**
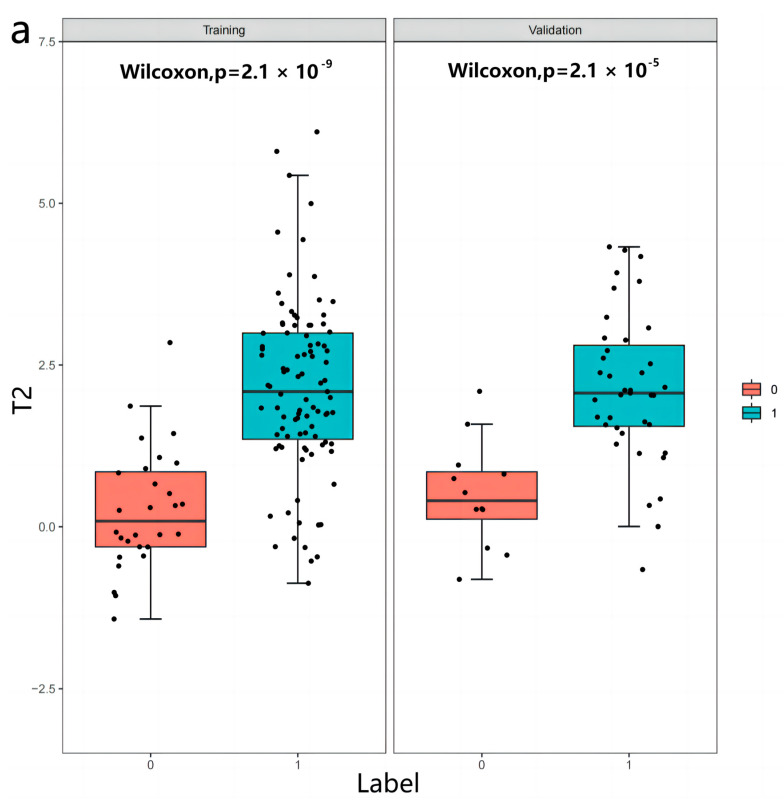

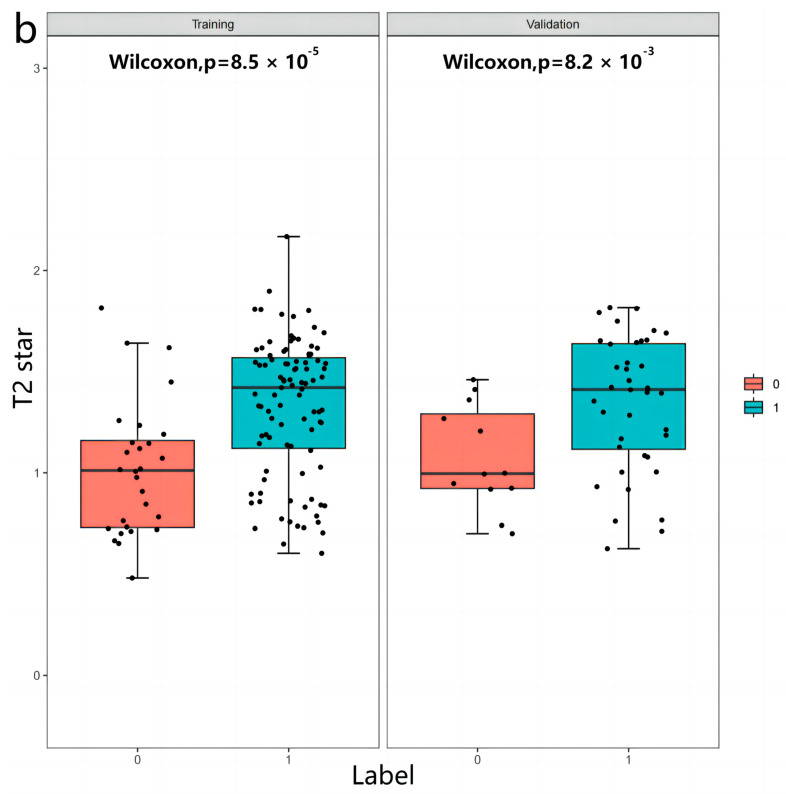

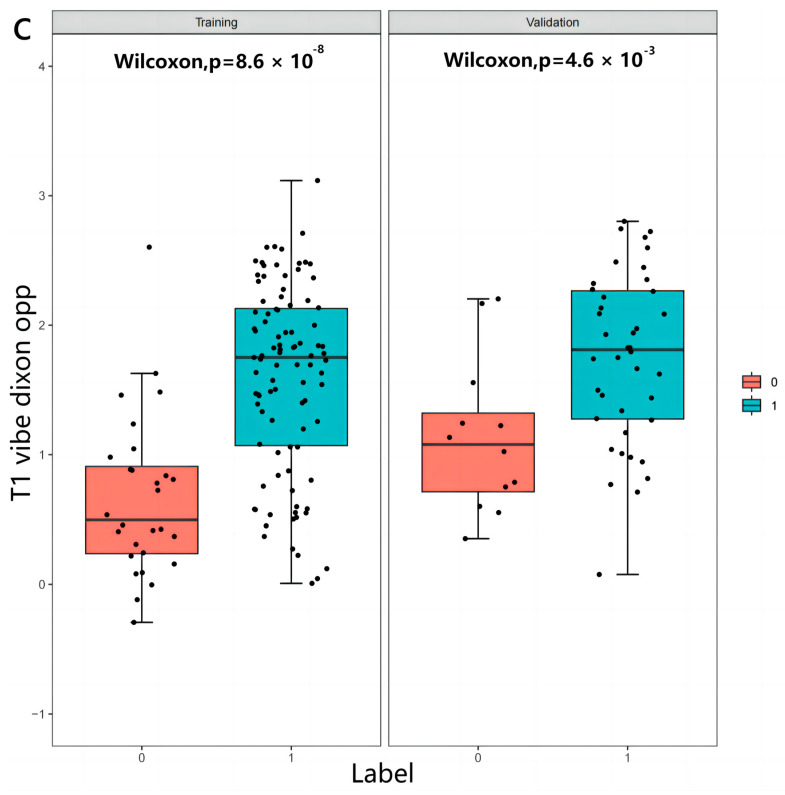

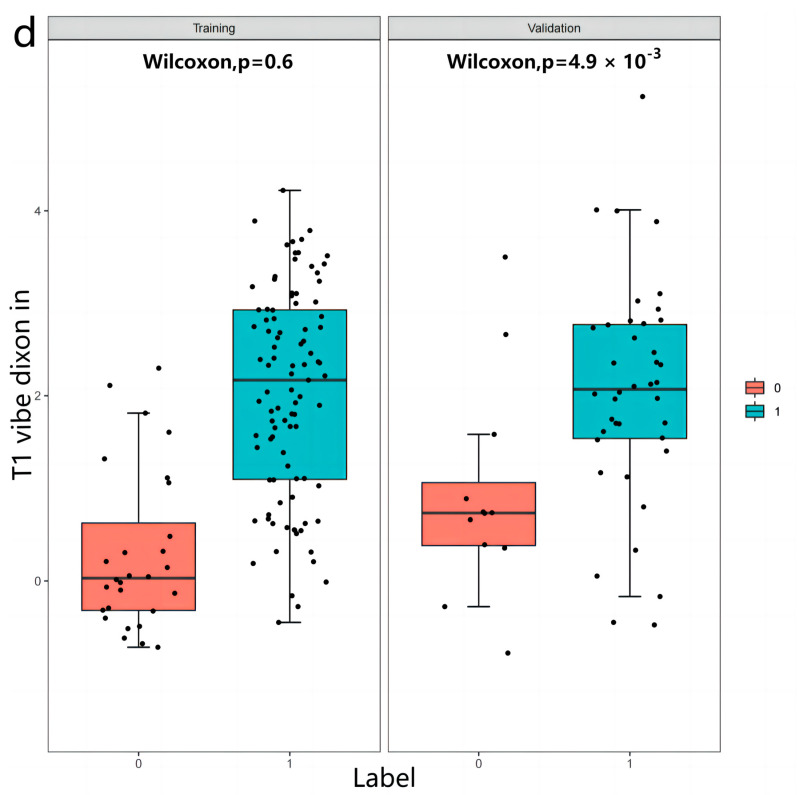

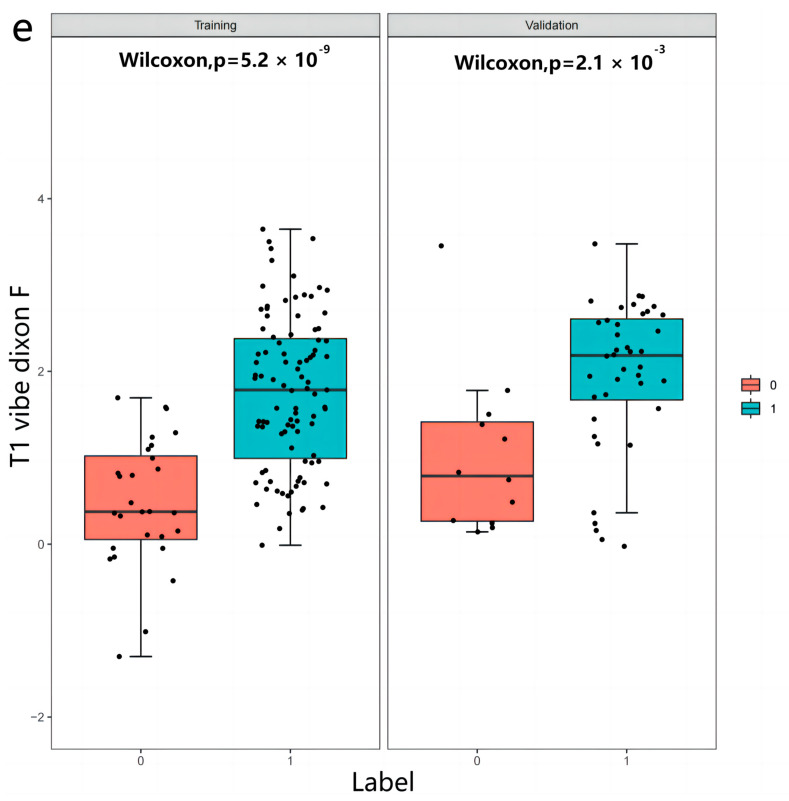

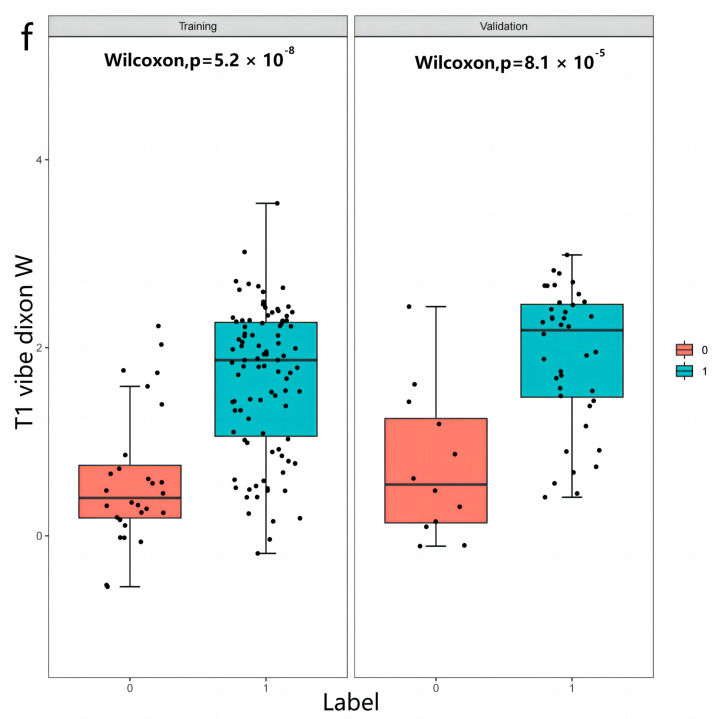
(**a**–**f**) represent the Rad score distributions of the training group and the validation group in the T2, T2*, T1 vibe dixon opp, T1 vibe dixon in, T1 vibe dixon F, and T1 vibe dixon W models, respectively. Box plots showed significant differences in Rad scores between Label 0 (α- genome) and Label 1 (β- genome) in different models (*p* < 0.05).

After the first mRMR screening, the best 20 features were retained for each image sequence. Then, LASSO was used to reduce the dimension (Figure 3), and a total of 47 optimal omics features were finally screened out. The optimal image features of different models and the nomogram of the joint model are shown in Figure 4. Tested using the Hosmer–Lemeshow test, all models had good fit (*p* > 0.05, Table A1, Appendix A). The T2 model was used as the optimal imaging model to construct a joint model with the clinical model. The joint model ROC is shown in Figure 2a,b. It can be seen in Figure 2b that the AUCs of the T2 model and the joint model validation group were 0.88 (95%CI = 0.78−0.99) and 0.91 (95%CI = 0.81−1.00), respectively. The ROCs of different radiomics models are shown in Figure 2c,d. The accuracy, sensitivity, and specificity results for different omics models and combined models are shown in Table 4. According to Table 4, the predictive performance of the joint model was higher than that of any single omics model or clinical model. The calibration curves and decision curves of the clinical model, the T2 model, and the joint model are shown in Figure 5. The Rad score distributions of different radiomics models are shown in the box plots in Figure 6.

### 3.3. Inter-Observer and Intra-Observer Reproducibility of Radiomics Feature Extraction

The intra-observer ICC calculated based on the two measurements for observer A ranged from 0.977 to 0.995, with *p* < 0.001. Inter-observer agreement based on two-observer measurements ranged from 0.933 to 0.986, with *p* < 0.001. The results show that the intra-observer and inter-observer feature extraction had good consistency. 

## 4. Discussion

Today, traditional prenatal testing remains the standard obstetric method for the prevention of thalassemia. When non-directive genetic counseling and supportive follow-up are required, traditional testing can provide critical information about fetal status for women and couples with high-risk pregnancies [1]. However, as previously described, to determine the TM genotypes of TM patients, standard detection methods require peripheral blood sampling, amniocentesis, and CVS, which are invasive, while CVS and amniocentesis are not only invasive but are also associated with high abortion risks (1/200–400 and 1/100–200, respectively) [9,10]. Radiomics methods have great potential in gene expression and typing, which has been reported in some studies on the application of radiomics [13,16,21,22]. In this study, a multiparametric radiomics method based on liver MRI was developed and validated for predicting α-and β-genotypes in TM patients. Although the radiomics models constructed in this study do not currently provide 100% accurate predictions of TM patient α- and β-genes and although different radiomics models have certain differences in terms of predictive efficiency with respect to α- and β-genes in TM patients, they all showed good predictive efficiency in the current study samples. 

For clinical factors, univariate and multivariate logistic regression analysis showed that age and ALB could be used as independent predictive factors, which indicated that age and ALB were associated with genotyping A and B to a certain extent. It is well known that the vast majority of TM patients, especially transfusion-dependent TM patients, need to receive transfusion therapy regularly or irregularly [23], so liver T2* value, blood routine, serum ferritin, and other clinical data will obviously have great volatility and unreliability. Therefore, the data for liver T2* values, blood routine tests, and serum ferritin were not included in the model and were only presented as baseline data.

To the best of our knowledge, this is the first study to predict genes in TM patients by constructing an organ MRI radiomics model. Different radiomics features can describe or reflect different information. For example, the first-order features can quantitatively describe the voxel distribution in the image. The characteristics of the gray level co-occurrence matrix can reflect the homogeneity and heterogeneity of lesions. The features of the gray run-length matrix can reflect information such as directionality and roughness of image texture [24]. This study was preliminary and exploratory, so we only selected one organ for the study. For image feature extraction, AK software was used to extract a total of 1316 features, including first-order, second-order, and higher-order features, which can be selected in the software for in-depth exploration.

Among the constructed radiomics models, the T2, T1 vibe dixon opp, T1 vibe dixon in, T1 vibe dixon F, and T1 vibe dixon W omics models showed high predictive performance—higher than clinical models. The T2 model had the highest predictive efficiency, and it showed a higher predictive efficiency when combined with the clinical model. (The AUC, sensitivity, and specificity of the training group were improved.) After constructing the joint model, the specificity of the predictive performance of the validation group was significantly reduced. It was believed that this was due to the low specificity and volatility of the clinical features themselves. The T2* radiomics model showed only moderate predictive performance. This may be related to the fact that functional imaging based on T2*/R2* technology is mostly used for the quantitative diagnosis of liver iron content [25], while it does not have obvious advantages for the heterogeneity of liver tissue and image resolution. 

Our study has the following limitations. First of all, as a preliminary exploration, this study was only based on the sample data of two research institutions for analysis, and there was no model validation based on more central data. Secondly, our study only selected the transverse MRI images of the largest liver level for analysis and did not cover the entire liver. However, NG et al. [26] showed that texture analysis with maximum cross-sectional area can be used as an alternative to overall texture analysis in conducting omics analysis. Thirdly, although 175 patients were included in this study, the sample size was reduced after the classification. The final constructed model was only used for a preliminary discussion in the study. Future work could be undertaken for single sequences and could include large samples for more precise model construction. Finally, most of the data in this study were for children or adults, and the fetal data set included only five cases. Our preliminary exploration only concerned the α-/β-monotype and did not involve gene analysis of other types (including complex and subtypes).

Despite these limitations, this study demonstrates that the radiomics model is very reliable for α- and β-genotyping in patients with TM. It is hoped that this study will help promote the non-invasive examination of TM patients by MRI technology and even provide ideas for more non-invasive genetic examinations. Future work will focus on the validation of this model and the construction of more gene prediction models to improve the diagnosis of different genotypes and their subtypes, as well as the construction of genotyping models based on fetal MRI radiomics to achieve clinical conversion of non-invasive gene testing.

## Figures and Tables

**Table 1:** Clinical baseline measurement data of α-genome and β-genome patients.

Variables	α-Genome (*n* = 40)		β-Genome (*n* = 135)	
	M	Interquartile Range	M	Interquartile Range
Liver T2 star (ms)	2.48	1.523–4.09	1.24	0.98–1.8
Serum ferritin (mg/mL)	474.86	311.478–895.198	3918.495	2343.943–6937.428
WBC (10^9^/L)	6.59	5.36–8.82	7.13	5.388–10.79
RBC (10^12^/L)	4.46	3.690–4.880	3.635	3.11–4.128
HGB (g/L)	88	79.3–101.2	92.3	82.925–109.975
PLT (10^9^/L)	300.1	222.3–348	263.2	181.525–426.925
NEU percentage (%)	0.574	0.459–0.63	0.473	0.344–0.579
LYM percentage (%)	0.35	0.295–0.418	0.381	0.314–0.504
MONO percentage (%)	0.061	0.057–0.079	0.077	0.061–0.101
EO percentage (%)	0.015	0.012–0.027	0.023	0.012–0.039
BISO percentage (%)	0.004	0.003–0.007	0.004	0.003–0.007
NEU (10^9^/L)	3.99	2.62–5.27	3.61	2.148–5.59
LYM (10^9^/L)	2.36	1.66–2.83	2.69	1.903–4.115
MONO (10^9^/L)	0.45	0.31–0.59	0.51	0.343–1.01
EOS (10^9^/L)	0.12	0.07–0.18	0.155	0.073–0.365
BASO (10^9^/L)	0.03	0.01–0.05	0.03	0.02–0.06
MCV (fl)	73.39	67.6–75.5	81.22	74.925–85.225
MCH (pg)	20.37	19–21.55	26.58	24.543–28.365
MCHC (g/L)	278	269.9–293	330.25	322.775–339.5
HCT (%)	0.316	0.278–0.35	0.283	0.247–0.335
RDWCV (%)	0.25	0.23–0.3	0.19	0.14–0.25
PDW (%)	0.16	0.14–0.19	0.16	0.11–0.18
PCT (%)	0.31	0.195–0.44	0.231	0.16–0.392
MPV (fl)	9.5	8.09–12.1	8.845	7.773–10.05

Note—Liver T2 star = Liver T2*. WBC = White blood count. RBC = Red blood cell. HGB = Hemoglobin. PLT = Platelets. NEU = Neutrophile granulocyte. LYM = Lymphocyte. MONO = Monocyte. EOS = Eosinophils. BASO = Basophil. MCV = Mean corpuscular volume. MCH = Mean corpuscular hemoglobin. MCHC = Mean corpuscular hemoglobin concentration. HCT = Hematocrit. RDWCV = Red blood cell volume distribution width. PDW = Platelet distribution width. PCT = Plateletocrit. MPV = Mean platelet volume.

**Table 2:** Univariate logistic regression analysis of the clinical characteristics.

Variable	Odds Ratio	Lower	Upper	*p*-Value
Age	0.904	0.857	0.954	<0.001
Gender	1.42	0.607	3.325	0.419
TBiL	0.994	0.982	1.007	0.366
DBiL	1.003	0.975	1.031	0.849
IBil	0.985	0.967	1.004	0.131
DB versus TB ratio	25.277	0.237	2695.483	0.175
ALB	0.801	0.681	0.942	0.007
GLO	0.967	0.889	1.053	0.44
A versus G ratio	1.096	0.29	4.137	0.893
GGT	1.005	0.99	1.019	0.538
TBA	1.095	1	1.198	0.05
AST	1.029	0.996	1.062	0.088
ALT	1.021	0.997	1.044	0.081
AST versus ALT ratio	0.604	0.352	1.038	0.068
PA	0.993	0.984	1.003	0.167
CHE	1	1	1	0.397
UREA	1.023	0.714	1.465	0.901
CREA	0.901	0.834	0.973	0.008
RBP	0.971	0.921	1.023	0.27
HCO_3_	0.953	0.801	1.135	0.591
Ccr	0.994	0.97	1.018	0.611
CysC	3.603	0.142	91.551	0.437

Note—TBiL = Total Bilirubin. DBiL = Direct Bilirubin. IBil = Indirect Bilirubin. DB versus TB ratio = Direct Bilirubin versus total Bilirubin ratio. ALB = Albumin. GLO = Glutamate Oxidase. A versus G ratio = Albumin versus Glutamate Oxidase ratio. GGT = Gamma-Glutamyl Transpeptidase. TBA = Total bile acids. AST = Aspartate amino Transferase. ALT = Alanine amino Transferase. AST versus ALT ratio = Aspartate amino Transferase versus Alanine amino Transferase ratio. PA = Prealbumin. CHE = Cholinesterase. CREA = Creatinine. RBP = Retinol-binding Protein. Ccr = Creatinine clearance rate. CysC = Cystatin C.

**Table 3:** Multivariate logistic regression analysis of the clinical characteristics.

Variable	Odds Ratio	CI.95%	*p*-Value
Age	0.939	[0.896;0.983]	0.009
ALB	0.831	[0.716;0.964]	0.044
TBA	1.064	[0.989;1.144]	0.107
CREA	0.936	[0.871;1.005]	0.14

Note—CI.95% = 95% confidence interval. ALB = Albumin. TBA = Total bile acids. CREA = Creatinine.

**Table 4:** Predictive efficacy of radiomics model, clinical model, and combined model.

Model	TP	TN	FP	FN	Accuracy	Sensitivity	Specificity	Pos. Pred. Value	Neg. Pred. Value
T2 test	35	10	2	5	0.865	0.875	0.833	0.946	0.667
T2 star test	26	8	4	14	0.654	0.65	0.667	0.867	0.364
T1 vibe dixon opp test	32	6	6	8	0.731	0.8	0.5	0.842	0.429
T1 vibe dixon in test	35	4	8	5	0.75	0.875	0.333	0.814	0.444
T1 vibe dixon F test	32	8	4	8	0.769	0.8	0.667	0.889	0.5
T1 vibe dixon W test	35	8	4	5	0.827	0.875	0.667	0.897	0.615
Clinics test	32	5	7	8	0.712	0.8	0.417	0.821	0.385
Combine test	36	8	4	4	0.846	0.9	0.667	0.9	0.667

Note—TP = True positive; TN = True negative; FP = False positive; FN = False negative; Accuracy = (TP + TN)/(TP + FN + FP + TN); Sensitivity = TP/(TP + FN); Specificity = TN/(TN + FN); Pos. Pred. Value = Positive predicative value = TP/(TP + FP); Neg. Pred. Value = Negative predicative value = TN/(TN + FN).

## Data Availability

The datasets generated during and/or analyzed during the current study are not publicly available but are available from the corresponding author on reasonable request.

## Appendix A

**Table A1:** Results of the Hosmer–Lemeshow tests for different radiomics models.

Radiomics Models	χ^2^	*p*-Value
T2 train	5.244	0.731
T2 test	3.695	0.884
T2 star train	5.354	0.719
T2 star test	7.998	0.434
T1 vibe dixon opp train	12.58	0.127
T1 vibe dixon opp test	13.666	0.091
T1 vibe dixon in train	5.244	0.731
T1 vibe dixon in test	3.695	0.884
T1 vibe dixon F train	5.354	0.719
T1 vibe dixon F test	7.998	0.434
T1 vibe dixon W train	12.58	0.127
T1 vibe dixon W test	13.666	0.091
